# UNDERSTANDING DEMENTIA DISCOURSE DURING ALZHEIMER’S AWARENESS MONTH IN CANADA: FIRST INSIGHTS FROM A TWITTER STUDY

**DOI:** 10.1093/geroni/igac059.2782

**Published:** 2022-12-20

**Authors:** Juanita-Dawne Bacsu, Megan E O’Connell, Allison Cammer, Mehrnoosh Azizi, Soheila Ahmadi, Karl Grewal, Shoshana Green, Corinne Berger

**Affiliations:** Thompson Rivers University, Kamloops, British Columbia, Canada; University of Saskatchewan, Saskatoon, Saskatchewan, Canada; University of Saskatchewan, Saskatoon, Saskatchewan, Canada; University of Saskatchewan, Saskatoon, Saskatchewan, Canada; University of Saskatchewan, Saskatoon, Saskatchewan, Canada; University of Saskatchewan, Saskatoon, Saskatchewan, Canada; University of Saskatchewan, Saskatoon, Saskatchewan, Canada; University of Saskatchewan, Saskatoon, Saskatchewan, Canada

## Abstract

Twitter has become a key platform for public health campaigns, ranging from mental health awareness week to diabetes awareness month. However, there is a paucity of knowledge about how Twitter is being used to support health campaigns, particularly for Alzheimer’s Awareness Month. This presentation aims to: 1) identify how Twitter was used to share dementia discourse during Canada’s Alzheimer’s Awareness Month in January; and 2) explore actions to enhance dementia awareness using Twitter for future Alzheimer’s Awareness Month campaigns. Tweets were collected from Twitter using the Twint application in Python from January 1 to January 31, 2022. Filters were used to exclude irrelevant tweets (5,820), and the remaining 1,289 tweets were exported to Excel. Tweets were divided among eleven coders and analyzed using inductive thematic analysis. Analysis revealed four main themes: dementia education and advocacy; fundraising and promotion; sharing experiences of dementia; and opportunities for future actions such as collaborative partnerships and educational tweets to correct stigmatizing language and dementia stereotypes. Increased educational content, collaborative partnerships, and evidence-informed research are essential to enhancing dementia awareness strategies on Twitter during Alzheimer’s Awareness Month in Canada. Further research is needed to develop, implement, and evaluate methods to improve dementia awareness on Twitter during Alzheimer’s Awareness Month and beyond.

